# Complete mitochondrial genome of the peninsula cooter (*Pseudemys peninsularis*, Testudines: Emydidae) in Korea

**DOI:** 10.1080/23802359.2022.2107463

**Published:** 2022-08-04

**Authors:** Dayeon Chung, Jaehong Park, Seungju Cheon, Seung-Min Park, Ha-Cheol Sung, Dong-Hyun Lee

**Affiliations:** a School of Biological Sciences and Biotechnology Graduate School, Chonnam National University, Gwangju, Korea; b Research Center of Ecomimetics, Chonnam National University, Gwangju, Korea; c Department of Biological Sciences, College of Natural Sciences, Chonnam National University, Gwangju, Korea

**Keywords:** *Pseudemys peninsularis*, Emydidae, mitochondrial genome

## Abstract

The complete mitochondrial genome of *Pseudemys peninsularis* in Korea was sequenced and characterized. The mitochondrial genome is constituted of 37 genes (13 protein-coding genes, 22 transfer RNA genes, and two ribosomal RNA genes) and a noncoding region. Phylogenetic analysis based on the 13 protein-coding gene sequences showed that *P. peninsularis* has closer relationship with *Chrysemys picta* than *Trachemys scripta elegans.* This is the first case for a complete mitochondrial genome from *P. peninsularis*, which will provide information for biogeographical studies and management plans for invasive species.

The peninsula cooter (*Pseudemys peninsularis* Carr, 1938) is spread throughout the Florida peninsula. It ranges from north along the Atlantic coast to the mouth of the St. John’s River (Seidel and Powell 1998). As the pet market expands, the influx of invasive species including turtles to Korea has been increasing (Koo et al. 2020). This increase causes many problems such as disturbing the domestic ecosystem, competition, and hybridization with native turtles. Hybridization with a closely related native species is critical and can increase the rate of infertility (Burke and Arnold 2001). The peninsula cooter has not been an invasive species yet, but it is possible that it will be designated as an invasive species soon (Lee et al. 2016). However, the survey for the invasive species is still insufficient. Furthermore, the complete mitochondrial genome of peninsula cooter has not been identified, though only a portion of its genetic information has been known (Spinks et al. 2013). In this study, we sequenced the complete mitochondrial genome of *Pseudemys peninsularis*, and this data can help phylogenetic studies and the management for the invasive species.

The *P. peninsularis* specimen was collected from Gwangju (35° 7′ 35.17“N, 126° 52′ 23.16“E), Korea, and the total genomic DNA was extracted from the tail using the DNeasy Blood and Tissue kit (Qiagen, Valencia, CA) according to the manufacturer’s protocol. The extracted DNA sample was deposited at the Museum of Wildlife, located in Research Center of Ecomimetics, Chonnam National University, Korea (Specimen accession number: 2021-RCE-PP001; shcol2002@jnu.ac.kr). The mitochondrial genome was analyzed using Illumina HiSeq X Ten platform (Illumina, San Diego, CA), which was performed by Macrogen (Seoul, Korea). Raw sequence data were checked by FastQC, and adaptor trimming and quality filtering were performed by Trimmomatic (Andrews 2010; Bolger et al. 2014). Subsequently, *de novo* assembly was conducted using SPAdes and the filtered reads were aligned using BLAST (Altschul et al. 1990; Bankevich et al. 2012). Finally, the complete sequence was annotated using MITOS2 web server (Bernt et al. 2013).

The complete mitochondrial genome of *P. peninsularis* is 16,754 bp in length deposited in GenBank (Accession number: OM935748), and contains 13 protein-coding genes, 22 transfer RNA (tRNA) genes, two ribosomal RNA (rRNA) genes, and a putative long non-coding control region. 12 protein-coding genes, 14 tRNA genes, and two rRNA genes are encoded in heavy strand, whereas one protein-coding gene (NADH dehydrogenase subunit 6) and eight tRNA genes in light strand. The nucleotide composition of the *P. peninsularis* mitochondrial genome (A = 34.5%, T = 26.6%, C = 25.9%, and G = 12.9%) is similar to that of *T. scripta elegans* from Korea (MW019443; A = 34.3%, T = 27.0%, C = 25.9%, and G = 12.9%), *Mauremys sinensis* from China (KC333650; A = 33.9%, T = 26.3%, C = 26.6%, and G = 13.2%), *M. reevesii* from Korea (KJ700438; A = 34.1%, T = 27.0%, C = 26.1%, and G = 12.8%), and *Chrysemys picta* from USA (AF069423; A = 34.4%, T = 26.8%, C = 25.9%, and G = 12.8%). The sequence of *P. peninsularis* has higher similarity with that of *C. picta* (93%) than other turtles including *T. scripta elegans* (90%), *M. sinensis* (81%), and *M. reevesii* (81%).

To investigate the phylogenetic position of *P. peninsularis*, the 13 protein-coding gene sequences of 14 species in *Testudines* were extracted from GenBank and the phylogenetic tree was constructed using MEGA X software (Figure 1; Kumar et al. 2018). Specifically, the sequences were aligned using MUSCLE algorithm and the phylogenetic tree was made using maximum likelihood method and GTR + G + I model with 1000 bootstrap replicates (Waddell and Steel 1997; Edgar 2004). GTR + G + I substitution model was selected as the best model by MEGA X. In agreement with sequence identity data, *P. peninsularis* is closer with *C. picta* than *T. scripta elegans*. But *P. peninsularis* is completely separated from *C. picta*. These data provide the information on the complete mitochondrial genome of *P. peninsularis* for the first time, and can contribute to further studies on biodiversity and management of *P. peninsularis* which is an invasive species in many countries including Korea.

**Figure 1.**
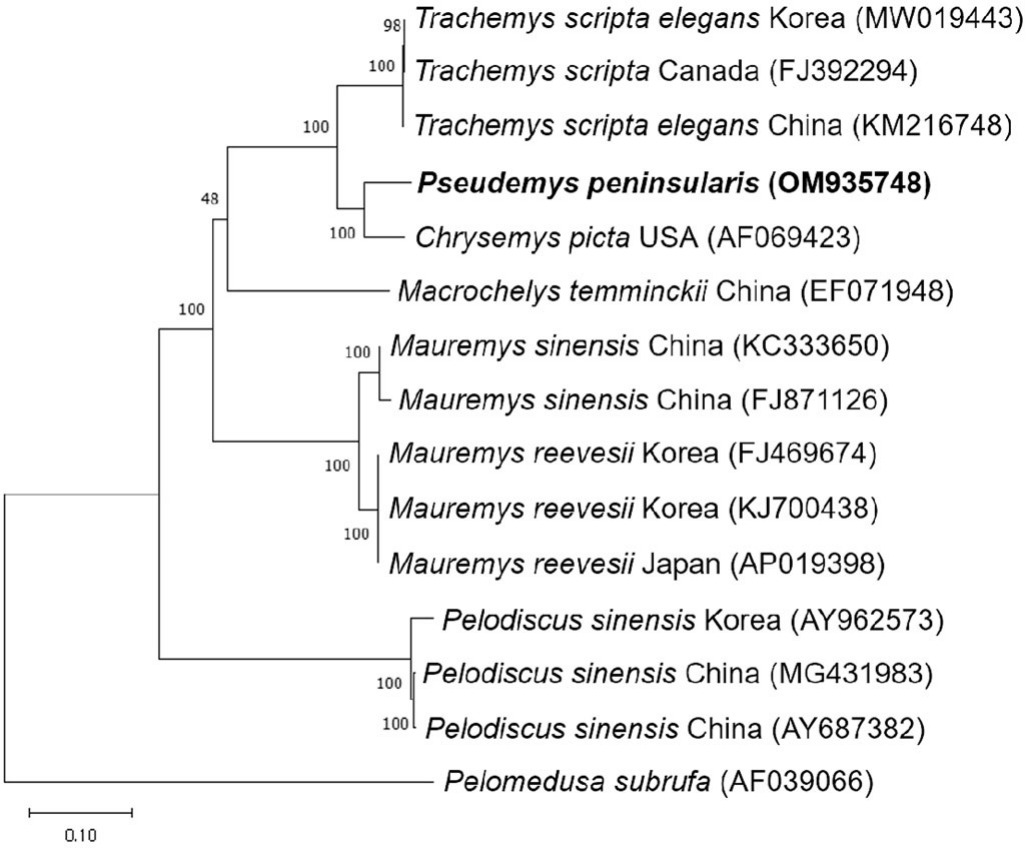
Phylogenetic tree of *Pseudemys peninsularis* and other related species based on 13 protein-coding gene sequences. Phylogenetic analysis was performed using MEGA X software. GenBank accession numbers of each mt genome sequence are given in the bracket after the species name, and the bootstrap value based on 1000 replicates is represented on each node. *Pelomedusa subrufa* was used as outgroup to root the tree.

## Data Availability

GenBank accession number from the complete mitochondrial genome of *Pseudemys peninsularis* (OM935748) has been registered with the NCBI database (https://www.ncbi.nlm.nih.gov/OM935748). The associated BioProject, BioSample, and SRA accession numbers are PRJNA813977, SAMN26520677, and SRR18578382, respectively.
